# Efficacy of Probiotics in Prevention of Migraine Attacks in Children: A Randomized Clinical Trial Study

**DOI:** 10.22037/ijcn.v17i4.39598

**Published:** 2024-03-12

**Authors:** Hassan BAZMAMOUM, Bentolhoda KESHTKARSOHI, Younes MOHAMMADI, Afshin FAYYAZI

**Affiliations:** 1 Department of Pediatric Gastroenterology, Besat Hospital, Hamadan University of Medical Sciences, Hamadan, Iran.; 2 Pediatrician, Department of pediatrics, Besat Hospital, Hamadan University of Medical Sciences, Hamadan, Iran.; 3 Department of Epidemiology, School of Public Health, Hamadan University of Medical Sciences, Hamadan, Iran; 4 Department of Pediatric Neurology, Hearing disorder research center, Hamadan University of Medical Sciences, Hamadan, Iran

**Keywords:** Migraine, Probiotics, Prevention, Children

## Abstract

**Objectives:**

Migraine is a chronic and joint disease in children. The results of previous studies on the effectiveness of probiotics in preventing migraine attacks in children have been controversial. This study aims to investigate the effect of probiotics on migraine prophylaxis in children.

**Materials & Methods:**

In this clinical trial study, 41 children aged 5 to 15 with migraine enrolled the study in two control and intervention groups. Children in the intervention group (18 children) received propranolol at a dose of 1 mg per kilogram of body weight daily in two divided doses along with a 250 mg Yomogi capsule daily for three months, and children in the control group (23 children), received propranolol along with placebo for three months. The study compared the frequency and duration of headache days, PedMIDAS criteria, and parental satisfaction between the two groups before treatment, as well as one month and three months post-treatment.

**Results:**

The number of headache days in both groups decreased over time, but in the intervention group, this decrease was more than the control group was statistically significant (P=0.045). The average PedMIDAS scale after treatment in the intervention group was 3.9 ± 3.8; in the control group, it was 8.4 ± 8.2, which was statistically significant (P=0.047). Parents’ satisfaction with the treatment was statistically significantly higher in the intervention group (94.4%) than in the control group (54.5%) (P=0.011). No significant drug complications were seen in any of the two groups.

**Conclusion:**

In children with migraine, adding probiotics to migraine treatment reduces the intensity and number of days of children’s headaches and increases the Parents’ satisfaction with the treatment.

## Introduction

Headaches are one of the most vital symptoms of various diseases in pediatrics. Two to six percent of all emergency visits for children and adolescents are due to headaches. After tension headaches, migraine is the second most common headache in children, occurring in different forms and requiring timely diagnosis and treatment (1, 2).

The brain of migraine patients is more sensitive to neurochemical changes due to genetic reasons, reducing the threshold of neuronal inflammation and the excitability of the trigeminal nerve. Consequently, trigeminal nerve sensory stimuli stimulate the somatosensory and limbic cortex (2, 3). The prevalence of migraine in children aged three to seven years is 1% to 3%, increasing to 8% to 23% in 15-year-olds (4).

Migraine prophylaxis treatments include non-pharmacological and pharmacological treatments. Non-pharmacological treatments include sleep pattern modification, diet, stress management, physical activity, and avoidance of irritants. Pharmacological prophylaxis treatments that are used more in children include beta-blockers, calcium channel antagonists, antiepileptics, and antidepressants (5).

Despite the efforts that have been made to find effective and safe preventive and therapeutic measures for migraine in children, existing drugs and non-pharmacological strategies are only partially useful (6, 7). Therefore, migraine control remains challenging.

Studies have indicated that the digestive and central nervous systems are interconnected, and one disturbance can impact the other. The term gut-brain axis refers to the mutual relationship between the central nervous and digestive system, involved in several diseases, including irritable bowel syndrome, helicobacter pylori infection, celiac disease, and headaches (8).

Glutamate, an excitatory neurotransmitter, is present in the intestinal nervous system and efferent nerves of the digestive system to the brain. It is effective in causing migraines through cortical spreading depression, central sensitization, and stimulating the trigeminovascular system. Previous research has shown that serotonin can alleviate migraine symptoms. Certain gut bacteria possess enzymes that boost the production of tryptophan metabolites, serving as building blocks for serotonin. Additionally, neurotransmitters like neuropeptide Y and calcitonin gene-related peptides are involved in the connection between the digestive system and brain disorders. (9).

On the other hand, probiotics are living and beneficial microorganisms play a significant role in improving the functioning of the digestive system. Most of the probiotics belong to the group of bifidobacteria or lactobacillus, which are part of the natural flora of the human intestine (10). The most crucial function of probiotics is the development, maturation, and regulation of the secretion-mucosal defense system. The first immunological effect of probiotics is probably to be located in the lymphatic tissue of the small intestine (11). In previous studies, the immune effects of probiotics have been shown. However, there is little direct evidence of the main mechanisms by which probiotics exert their effects (12). Probiotics improve migraine symptoms by affecting the immune system, and changing intestinal flora, and changing the vagus nerve signal and the level of some neurotransmitters. (13).

Many studies have been conducted on the effect of using probiotics in the prevention of chronic and episodic migraine. (11, 14, 15).

However, the findings could be more extensive and have mainly led to conflicting results. The majority of research has been carried out with adult participants, with only a limited number focusing on children. Considering the few studies conducted in children, as well as the limitations and contradictions in these studies, we decided to investigate the effect of probiotics on preventing migraine in children.

## Materials & Methods

 In this clinical trial study, forty-six children aged five to 15 with migraine who were referred to the pediatric neurology clinic of Besat Hospital in Hamadan, Iran, from 2021-2022 were studied. The inclusion criteria included diagnosis of migraine by a pediatric neurologist based on the criteria of the International Headache Society and age between five and 15 years. Exclusion criteria included suffering from other types of headaches, including chronic or drug-related headaches, suffering from inflammatory bowel diseases, suffering from neurological disorders, using antibiotics within three months before the study, using probiotics within two weeks before the study, contraindications to the use of propranolol such as asthma or diabetes, not consenting to participate in the study.

The children who entered the study were randomly divided equally into intervention and control groups using a random number table. Three patients in the control group and two in the intervention group were unwilling to continue cooperation and were excluded. Children in the intervention group (18 children) were treated for three months with one capsule of Yomogi 250 mg (Yomogi Company) along with propanol (Tolid Darou Company) at a dose of 1 mg/kg, and children in the control group (23 children) were treated with propranolol at a dose of 1 mg/kg (Tolid Darou Company) along with placebo for three months. Then, the number of headache days, headache duration, PedMIDAS disability severity scale, and parents’ satisfaction were compared between the two groups before, one month, and three months after treatment.

PedMIDAS and 3-point Likert questionnaire were used to collect data in addition to patient’s the demographic and clinical information questionnaire.

In the PedMIDAS questionnaire, using six questions, the number of days per month that the child’s performance was impaired at home, online classes, games, parties, and social activities due to headache was recorded, and based on the total scores obtained, the severity of disability caused by migraine headache was determined (16). A 3-point Likert questionnaire (satisfied, unaffected, and dissatisfied) was used to evaluate parents’ satisfaction with migraine treatment. 

To determine the sample size according to the results of Martami et al.’s study (15), considering the alpha of 5% and the power of 80%, as well as the difference in the migraine intensity score between the two groups, with a standard deviation of 2 using G power software, at least 18 patients for each group was considered.

Data analysis was conducted using SPSS statistical software, version 2. 

The significance level of the tests was considered less than 0.05.

This study was conducted with the approval of the Ethics Committee of the Hamadan University of Medical Sciences with the ethics code, the Deputy of Research and Technology ID IR.UMSHA.REC.1400.878, IRCT20160523028008N22. 

Besides, informed consent was obtained from all the patients or their parents.

## Results

In this study, forty-six children were enrolled in two equal groups. Three patients in the control group and two in the intervention group were unwilling to continue cooperation and were excluded. Both study groups had no statistically significant differences in demographic and clinical variables (Table 1). After the treatment, 17 (94.4%) parents of the intervention group were satisfied with the treatment of their children, while this rate was 54.5% in the control group, a statistically significant difference (P=0.011). In both groups, the number of headache attacks per month decreased, and in the intervention group, this decrease was statistically significantly higher than in the control group (Table 2). The two study groups did not have a statistically significant difference before the intervention regarding disability using the PedMIDAS scale. After the intervention, the paired t-test showed that the amount of disability in both groups decreased statistically significantly, and this decrease in the intervention group was significantly higher than the control group (Table 3).

No significant drug side effects were seen in any of the two groups.

**Table 1:** Patient characteristics in intervention and control groups

P-value	Intervention groups (n=18)	control groups ( n=23)	Variable
0.262	11(61.1%)	10(43.5%)	Gender(male)
0.327	8.9±3.1	7.9±2.4	age (years)
0.638	38.6±1.2	38.7±3.4	Age of headache onset (months)
1.00	1(6.6%)	2(8.7%)	History of previous illness
0.495	0	2(8.7%)	History of seizures
1.00	2(11.1%)	2(8.7%)	History of head injury
0.679	4(22.2%)	3(13%)	History of developmental problems
0.745	15(83.3%)	20(87%)	Family history of migraine
0.706	3(16.7%)	6(26.1%)	History of drug treatment
0.573	2(11.1%)	1(4.3%)	History of hospitalization
0.215	11(61.1%)	9(39.1%)	Change in sleep pattern
1.00	11(61.1%)	13(56.5)	Decrease in patient performance
0.524	12(66.7%)	12(52.2%)	Interference with academic activity
0.539	9(50%)	14(60.9%)	History of diagnostic imaging
0.585	10±5.3	8.8±7.7	Number of headache attacks in the month before treatment
1.00	3(16.7)	3(13%)	Relationship with menstruation
_	18(100%)	23(100%)	Common type of migraine
0.091	4(22.2%)	11(47.8%)	Headache intensity mild, moderate)

**Table 2:** Number of headache attacks in two study groups at the beginning, one and three months after treatment

Control group	Intervention group		ANOVA		Number of attacks
Mean ± SD	Mean ± SD	Time	Time with group	Group	
F=57.5	Sig=0.001	ηp2= 0.59	The beginning of the study	10±5.3	8.8±7.7
F= 1.3	Sig=0.284	ηp2= 0.032	One month after treatment	2.5±2.3	2±1.1
F= 10.1	Sig=0.045	ηp2= 0.35	Three months after treatment	2±1.7	0.6±0.8

**Table 3:** PedMIDAS score before and after intervention in two groups

P(t-test)	Intervention groups ( n=18)	control groups (n=23)	Variable
	Mean ± SD	Mean ± SD	
0.589	24.9±23.2	21.5±17.6	Before intervention
0.047	3.9±3.8	8.4±8.2	After intervention
0.585	0.001	0.001	P(Paired- t- test)

## Discussion

Several studies have been conducted on the prophylactic effect of probiotics on patients with migraine, but no study was found on their effect on children with migraine.

This study’s findings showed that adding probiotics at a dose of 250 mg/day to propranolol treatment in children with migraine reduced the number of headache days, reduced the disability scale, and increased parental satisfaction with the treatment.

Several mechanisms have been explained for the effect of probiotics in treating and preventing neurological diseases, including their effect on the immune system, changing the intestinal flora, changing the vagus nerve signal, and also changing the level of some neurotransmitters (13, 17). Several reasons have been suggested for improving migraine headaches following the use of probiotics, including improving gut integrity (18), reducing the entry of lipopolysaccharides from the lumen into the systemic circulation due to balancing the intestinal bacteria and reducing Gram-negative bacteria (19), increasing serotonin (20) and helping to increase the emptying rate of stomach contents due to neuroimmune interaction (21). Previous studies have shown that changes in the microbiome affect serotonin neurotransmission in both peripheral and central nervous systems. In a meta-analysis study by Wallace et al. (22), which reviewed ten articles, probiotics were associated with increased serotonin and improved mood in patients. The study of Desbonnet et al. (23) showed that the consumption of probiotics increases the plasma level of the precursor of serotonin, i.e., tryptophan. According to the findings of Sajjadi et al.’s study (2021) conducted on an animal model, the consumption of probiotics can increase serotonin in mice (24). 

The clinical trial study by Martami et al. (15) was conducted on thirty-nine episodic and forty chronic migraine patients. Two probiotic capsules containing 14 strains were prescribed to patients for ten weeks. The results of the study showed that the consumption of probiotics can improve migraine headaches in both chronic and episodic types. According to Arianfar et al.’s study (11) in 2022, consuming more low-fat dairy products may reduce migraine attacks in children and adolescents.

According to Straube et al.’s study (25) in 2018, an observational study with a large sample size, the consumption of probiotics for eight weeks reduced the headache score of patients from 1.5 to 1.2 and reduced migraine attacks from two times per week to 1.4 times per week. Similarly, other complications related to migraines decreased significantly in these people. In 2010, a randomized controlled trial study on healthy volunteers showed that probiotics can improve the intestinal epithelial barrier by modulating the expression of tight junction proteins in the intestinal epithelium (12). Since probiotics can correct increased intestinal permeability and maintain intestinal barrier function, they may relieve migraine headaches by improving intestinal epithelial permeability. In another clinical study, forty patients with migraine headaches received several nutrients for three months, including vitamins, minerals, micronutrients, herbs, and probiotics. At the beginning of this trial, the participants had an average quality of life score of 38, which increased to 76 after treatment, and experienced a 60% reduction in headache attacks (26). In a clinical trial study of volunteers with migraine headaches, participants received two grams per day of multi-strain probiotic products for 12 weeks. The study’s results showed that the number of migraine headache days decreased by 67%, and almost no severe side effect of medication was observed (27).

However, the results of the study by Roos et al. in 2017 on 63 patients with migraine showed that, compared to placebo, probiotics cannot be effective in reducing the intensity and frequency of headaches (28). According to the opinion of Dai et al. in 2017, there is limited evidence of the positive effects of probiotics on migraine headaches and a need to conduct multiple clinical trial studies with a larger sample size (17).

According to the meta-analysis study by Naghibi et al. in 2019, which found only two studies eligible for review, there still needs to be a consensus regarding the use of probiotics in migraine headaches, and more studies are needed (13). In the meta-analysis study of Parohan et al., three clinical trial studies were selected after the literature review. Out of 179 patients with migraine, ninety-four people had received probiotics, and the results showed that the consumption of probiotics has no significant effect on the frequency and intensity of headaches in patients. (14) The reason for the difference in the results of the studies may be due to the difference in the type of probiotic consumed, the studied populations, the duration of the intervention, and the methods used to evaluate the response to the treatment.

## In Conclusion

The findings of this study showed that in children with migraine, adding probiotics to the treatment of migraine reduces the severity of headaches and the number of headache days and increases parental satisfaction with the treatment.

Probiotics are generally well-tolerated and have minimal side effects, suggesting they could be beneficial for children with migraines. However, further research is necessary before incorporating probiotics into their treatment regimen. 

## Author’s Contribution

Conception and design: Hassan Bazmamoun, Afshin fayyazi, Bentolhoda Keshtkar Sohi. 

Analysis and interpretation: Hassan Bazmamoun, Afshin fayyazi, Bentolhoda Keshtkar Sohi, Younes Mohammadi.

Data collection: Hassan Bazmamoun, Afshin fayyazi, Bentolhoda Keshtkar. 

Writing the article: Hassan Bazmamoun, Afshin fayyazi

Critical revision of the article: Afshin fayyazi, Hassan Bazmamoun. 

Final approval of the article: Afshin fayyazi

Statistical analysis: Bentolhoda Keshtkar, Younes Mohammadi

Overall responsibility: Afshin fayyazi

## Conflicts of Interest

The results of this study are not in conflict with the authors’ interests.
